# Integrated Bioinformatics Analysis to Screen Hub Gene Signatures for Fetal Growth Restriction

**DOI:** 10.1155/2023/3367406

**Published:** 2023-03-30

**Authors:** Jingjin Yang, Yuxin Liu, Minyue Dong

**Affiliations:** ^1^Women's Hospital, School of Medicine, Zhejiang University, Hangzhou, China; ^2^School of Medicine, Taizhou University, Taizhou, Zhejiang, China; ^3^Key Laboratory of Women's Reproductive Health of Zhejiang Province, Hangzhou, China; ^4^Key Laboratory of Reproductive Genetics, Ministry of Education, Zhejiang University, Hangzhou, China

## Abstract

**Background:**

Fetal growth restriction (FGR) is the impairment of the biological growth potential of the fetus and often leads to adverse pregnancy outcomes. The molecular mechanisms for the development of FGR, however, are still unclear. The purpose of this study is to identify critical genes associated with FGR through an integrated bioinformatics approach and explore the potential pathogenesis of FGR.

**Methods:**

We downloaded FGR-related gene microarray data, used weighted gene co-expression network analysis (WGCNA), differentially expressed genes (DEGs), and protein-protein interaction (PPI) networks to screen hub genes. The GSE24129 gene set was used for validation of critical gene expression levels and diagnostic capabilities.

**Results:**

A weighted gene co-expression network was constructed, and 5000 genes were divided into 12 modules. Of these modules, the blue module showed the closest relationship with FGR. Taking the intersection of the DEGs and genes in the blue module as pivotal genes, 277 genes were identified, and 20 crucial genes were screened from the PPI network. The GSE24129 gene set verified the expression of 20 genes, and CXCL9, CXCR3, and ITGAX genes were identified as actual pivotal genes. The expression levels of CXCL9, CXCR3, and ITGAX were increased in both the training and validation sets, and ROC curve validation revealed that these three pivotal genes had a significant diagnostic ability for FGR. Single-gene GSEA results showed that all three core genes activated “hematopoietic cell lineage” and “cell adhesion molecules” and inhibited the “cGMP-PKG signaling pathway” in the development of FGR. CXCL9, CXCR3, and ITGAX may therefore be closely associated with the development of FGR and may serve as potential biomarkers for the diagnosis and treatment of FGR.

## 1. Introduction

Fetal growth restriction (FGR), also knowns as intrauterine growth restriction (IUGR), means that the fetus cannot reach its biological growth potential and is a common complication of pregnancy [1]. It is usually used to describe fetuses whose estimated fetal weight or abdominal circumference is less than the 10th percentile for gestational age [2]. It is well known that FGR is a major cause of fetal, perinatal, and neonatal morbidity and mortality. Infants with FGR are prone to long-term health problems such as poor physical growth, metabolic syndrome, cardiovascular disease, neurodevelopmental disorders, and endocrine abnormalities [3].

The pathogenesis of FGR is related to maternal, fetal, placental, and genetic factors, among which placental insufficiency is the leading cause [4]. The placenta is a vital tissue that connects the mother to the fetus. If the placental blood perfusion is insufficient, the fetus suffers from chronic hypoxia and decreased growth rate [5]. Compared to normal controls, pregnancies with FGR (with or without preeclampsia) had smaller placental volumes and more excellent resistance to uterine blood flow [6]. Many types of research showed that insufficient chorionic trophoblast infiltration, defective maternal uterine artery remodeling, and placental inflammation are associated with inadequate placental perfusion [7–10].

Although there are many studies on the pathogenesis of FGR, its specific pathological mechanisms are still not fully elucidated. At present, with the rapidly developing microarray technology and high-throughput sequencing technology, bioinformatics is used to study the pathogenesis of FGR. In this research, we used WGCNA to explore the characteristics of the placental gene network associated with FGR and to identify novel biomarkers of FGR pathogenesis.

## 2. Materials and Methods

### 2.1. GEO Dataset Download and Process

**Figure 1:**
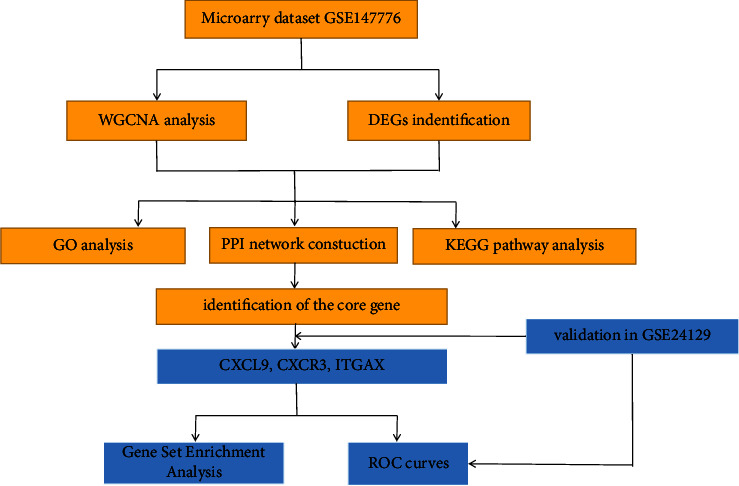
The flowchart of this study.

The workflow analysis is as follows (see Figure 1). Data were collected from the Gene Expression Omnibus (GEO) database (https://www.NCBI.nlm.gov/GEO). We used the keywords “fetal growth restriction” or “intrauterine growth restriction” to search for FGR or IUGR gene expression profiles from the database of GEO. The screening standards for this study were as follows. (1) The gene expression profiles must include a case group of patients with FGR or IUGR and a control group of normal pregnant women. (2) The tissue used for sequencing should be placenta. (3) For the WGCNA to be accurate, there should be at least 15 samples. (4) Datasets should contain either raw data or processed data, and these data should be microarray data. Finally, we selected GSE147776 and GSE24129 for further research analysis, GSE147776 as a discovery cohort and GSE24129 as a validated cohort. After downloading the normalized data, we filter the data to remove probes without corresponding annotations and take the maximum value for duplicate probes.

### 2.2. WGCNA

We used RStudio 4.1.3 software to process all data, in which co-expression networks were constructed using the WGCNA package [11]. We selected the top 5000 genes with median absolute deviation values for the WGCNA based on GSE147776. To exclude the outlier samples, the samples were clustered by hierarchical clustering analysis. To ensure scale-free topology, when the correlation coefficient threshold was used at 0.85, the soft-thresholding power was chosen to be 12 and the minimum module size was chosen to be 50. We defined 0.25 as the threshold of cutting height to merge the potentially similar modules. The expression of each module was calculated by module eigengenes (MEs), and the relationships between ME and clinical features were analyzed. Finally, we selected the module with a high coefficient of correlation with clinical features and selected the genes of this module for further analysis.

### 2.3. DEG Analysis

DEGs in the FGR and control groups were screened with the “limma” package [12]. The critical values for differential genes were taken as |log2 (fold change)| > 1.5 and *P* value <0.05. Using the Venn diagram program, overlapping genes of the WGCNA blue module genes and the DEGs were screened and visualized. These overlapping genes were identified as core genes.

### 2.4. Functional Enrichment Analysis of Hub Genes

Gene Ontology (GO) and Kyoto Encyclopedia of Genes and Genomes (KEGG) pathway enrichment analyses were performed for overlapping genes using the “clusterProfiler” R package [13]. Adjusted *P* value <0.05 was considered significantly.

### 2.5. PPI Network Construction and Hub Gene Identification

To construct a gene action network, 277 hub genes were mapped to the STRING database (https://string-db.org/). Then, we used the CytoHubba plugin for the base Cytoscape software (https://www.cytoscape.org/, version 3.9.1) to build protein interactions and visualize them, from which we selected the genes with the highest degree of connectivity as the central genes.

### 2.6. Hub Gene Expression Validation and Efficacy Evaluation

Validation of hub genes in the dataset GSE24129 downloaded from the GEO database was performed. The expression of core genes in FGR and normal control placental tissues was analyzed using the “ggplot2” package. Statistically significant differential genes were used for further ROC curve analysis. ROC curves were plotted, and the area under the curves (AUCs) were calculated using the “pROC” software package to assess the ability of the selected genes to discriminate between FGR and control groups [14].

### 2.7. Gene Set Enrichment Analysis

A gene set enrichment analysis (GSEA) was performed on individual hub genes separately in order to further explore the potential molecular functions of these genes in FGR. In the dataset GSE147776, we divided the samples into two groups in accordance with the median expression of the pivotal genes in the FGR and performed GSEA using the R package “clusterProfiler” with a *P* value <0.05 for the cutoff criterion.

## 3. Results

### 3.1. Information of Datasets

In accordance with the established search criteria, we found two datasets, GSE147776 and GSE24129. The specific information of the two datasets is shown in Table 1, and the clinical information of maternal and neonatal characteristics [15, 16] is presented in Table 2.

### 3.2. Weighted Co-Expression Network Construction and Key Module Identification

**Figure 2:**
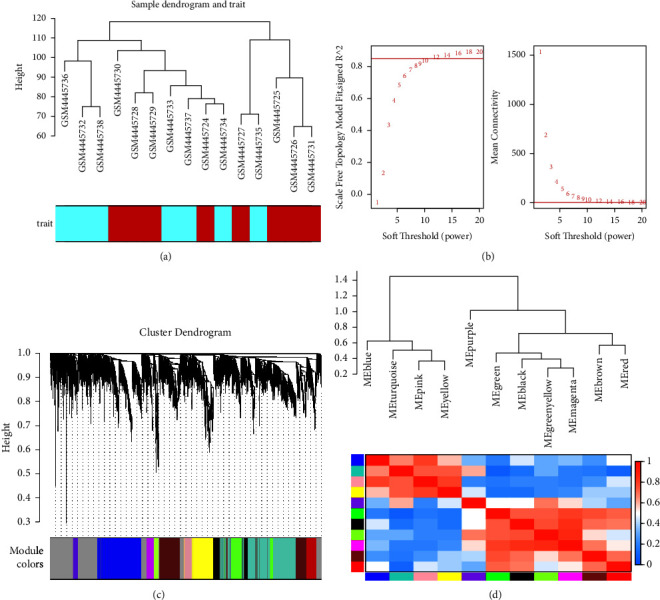
WGCNA of dataset GSE147776. (a) Cluster dendrogram of samples. (b) Determination of soft threshold power. (c) Clustering dendrogram of the top 5000 genes with the median absolute deviation value in GSE147776. (d) Module eigengene adjacency heatmap.

**Figure 3:**
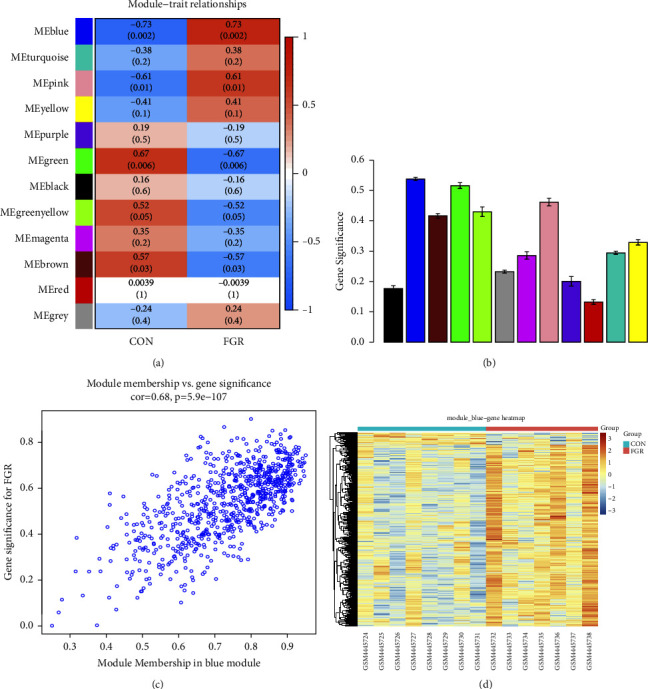
Module-trait correlation analysis. (a) Module-trait relationship heatmap (each cell contains correlation coefficients and corresponding *P* values). (b) Module significance values of co-expression modules associated with FGR (module significance values indicate the summary of gene significance of all genes in each module, and different colored columns indicate different modules). (c) Gene significance of FGR in blue modules. (d) Heatmap of genes in the blue module.

To find the most associated gene sets with the FGR trait, we used the WGCNA package to construct a gene co-expression network. We first examined genes and samples, then performed cluster analysis on samples to exclude outliers, and finally collected all 15 clinical samples from the GSE147776 dataset for analysis (see Figure 2(a)). In this dataset, when R2 of the spectrum structure of the scale-free network was used at 0.85, the soft threshold power is 12, ensuring that the network was approaching a scale-free topology (see Figure 2(b)). 12 co-expression modules were constructed by WGCNA (see Figure 2(c)). These modules were divided into 2 clusters (see Figure 2(d)). We drew a heat map of module-trait relationship to assess the correlation of all modules with FGR and found that the blue module had the highest positive correlation with FGR, so we selected this module for further analysis (see Figure 3).

### 3.3. DEGs and Hub Gene Identification

**Figure 4:**
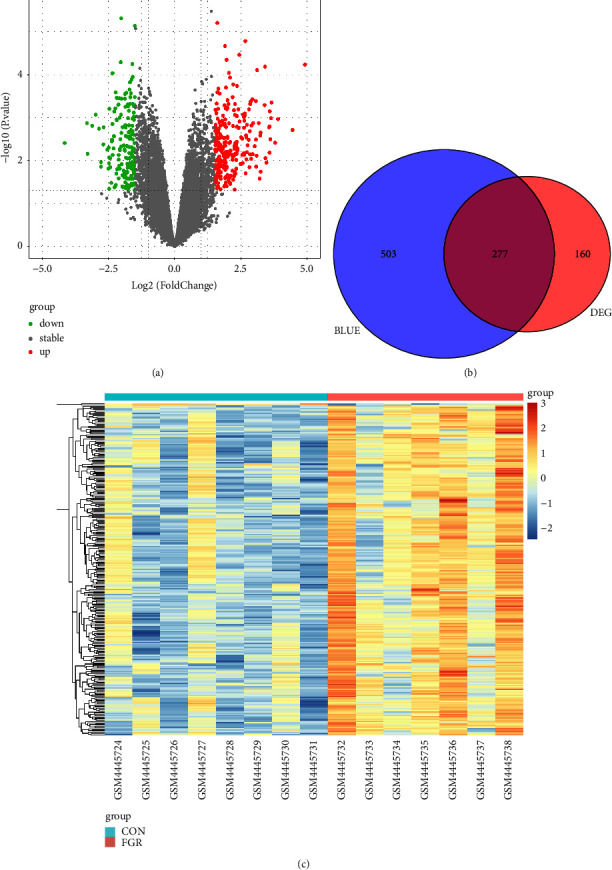
Identification of overlapping hub genes from DEGs and the blue modules of WGCNA. (a) Volcano plot of differentially expressed genes (DEGs) from GSE147776 normal and FGR samples. (b) Venn diagram representing overlapping genes in differentially expressed genes (DEGs) and the blue module of WGCNA. (c) Heatmap of overlapping hub genes.

In total, 437 DEGs have been identified in GSE147776, including 325 upregulated genes and 112 downregulated genes. The volcano plot of the DEGs is illustrated in Figure 4(a). We identified 277 candidate genes from the intersection of the DEGs and the WGCNA blue module genes in the Venn diagram (see Figure 4(b)). The heatmap of the extract hub genes is displayed in Figure 4(c).

### 3.4. GO and KEGG Analyses

**Figure 5:**
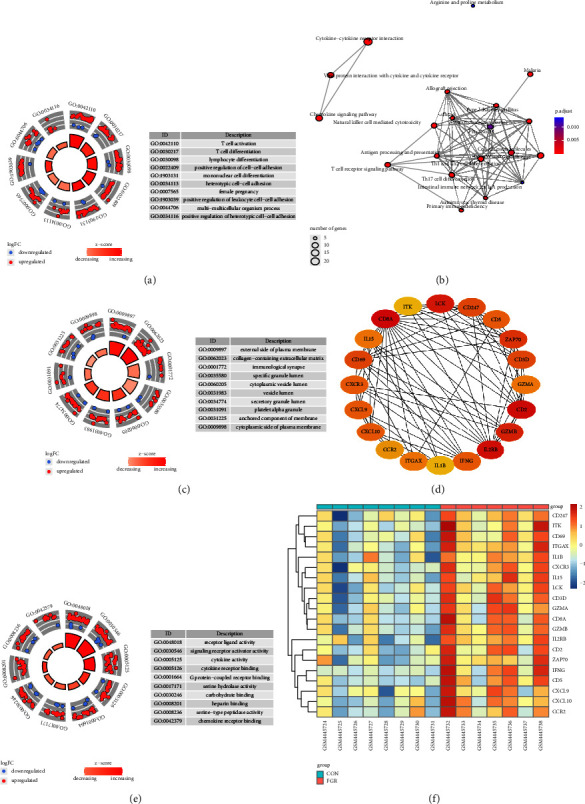
Functional analysis of the hub genes. (a) Biological process analysis. (b) KEGG pathway analysis. (c) Cell component (CC) enrichment analysis. (d) PPI network between 20 core genes. (e) Molecular function analysis. (f) Heatmap of 20 core genes.

The “clusterProfiler” package was used for GO function enrichment analysis to investigate the biological characteristics of 277 hub genes. In biological process, the hub genes were mainly enriched in the regulation of T cell activation, T cell differentiation, lymphocyte differentiation, and positive regulation of cell-cell adhesion (see Figure 5(a)). In cell component (CC), they were mainly enriched in the external side of plasma membrane, collagen-containing extracellular matrix, immunological synapse, and specific granule lumen (see Figure 5(c)). In molecular function, the hub genes were mainly enriched in the receptor ligand activity, signaling receptor activator activity, cytokine activity, and G protein-coupled receptor binding (see Figure 5(e)). In addition, KEGG enrichment analysis revealed the following pathways: cytokine-cytokine receptor interaction, hematopoietic cell lineage, graft-versus-host disease, and viral protein interaction with cytokine and cytokine receptor (see Figure 5(b)).

### 3.5. PPI Network Construction and Core Gene Analysis

For further study, we constructed a PPI network among 277 candidate genes in the STRING database and visualized the PPI network using Cytoscape software. Potential key genes were identified by the CytoHubba plugin (see Figure 5(d)). The top 20 genes in Hubba nodes were collected as pivotal genes. The heatmap of 20 hub genes is shown in Figure 5(f).

### 3.6. Core Gene Validation and Validity Assessment

**Figure 6:**
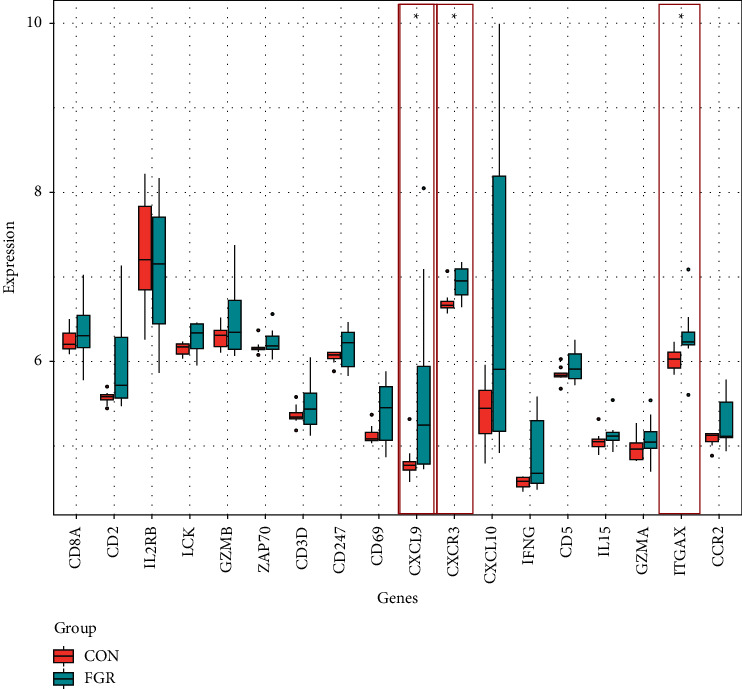
Validation of 20 core genes in dataset GSE24129.

**Figure 7:**
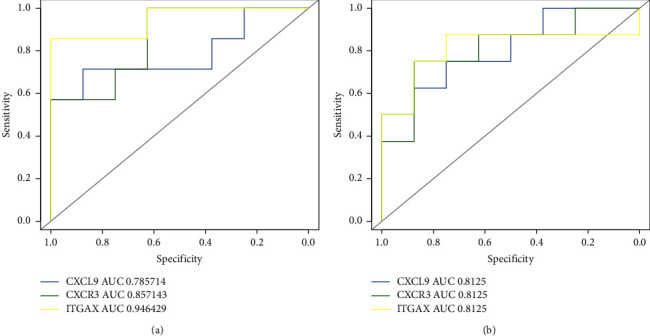
ROC curve of 3 hub genes (CXCL9, CXCR3, and ITGAX) in two datasets. (a) GSE147776. (b) GSE24129.

The extracted core genes were verified in the GSE24129 database, which revealed that CXCL9, CXCR3, and ITGAX were significantly increased in the expression of placental tissue from FGR patients (see Figure 6). These genes' expression levels consistently matched their expression in GSE147776. Additionally, ROC curve was plotted and AUC was measured to distinguish FGR from the control group; in dataset GSE147776, the AUC of CXCL9 was greater than 0.78, and the AUCs of CXCR3 and ITGAX were both greater than 0.85, while in GSE24129, the AUCs of all true pivotal genes were above 0.8 (see Figure 7).

### 3.7. Gene Set Enrichment Analysis

**Figure 8:**
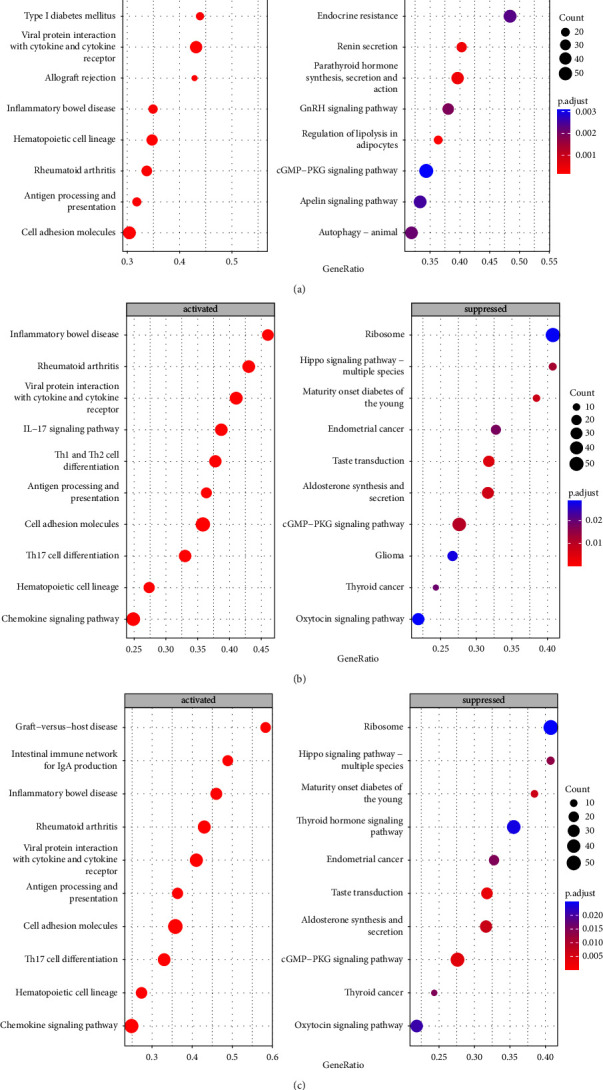
Gene set enrichment analysis (GSEA). (a) The KEGG pathway in CXCL9 (highly expressed). (b) The KEGG pathway in CXCR3 (highly expressed). (c) The KEGG pathway in ITGAX (highly expressed).

To analyze the potential molecular mechanisms of the core genes CXCL9, CXCR3, and ITGAX in FGR, we used single-gene GSEA to analyze the KEGG pathway. We found that “hematopoietic cell lineage” and “cell adhesion molecules” were activated in the high-expression groups of each of CXCL9, CXCR3, and ITGAX, while “cGMP-PKG signaling pathway” was inhibited (see Figure 8), suggesting that these pathways may be closely related to the development of FGR.

## 4. Discussion

FGR is a significant cause of stillbirth, neonatal mortality, and short- and long-term morbidity [1]. To date, there are no good treatment options for FGR except for iatrogenic preterm birth [17]. The most common factor for FGR is placental dysfunction; accordingly, the samples selected for this study were all placental tissues, excluded samples with combined preeclampsia.

WGCNA can be used to efficiently integrate data on gene expression and trait, explore the characteristics of gene networks, and identify regulatory pathways and potential biomarkers associated with complex diseases [11]. In the present study, based on WGCNA, the blue module (780 genes) was identified to be associated with FGR, and an additional 437 genes were identified by differential gene analysis. Interestingly, 277 of these intersecting genes were enriched in immune cell activation, differentiation, and regulation of cell adhesion, suggesting that the placenta exhibits inflammatory and immune abnormalities. Studies have found that placental inflammation is associated with intrauterine growth restriction [10, 18, 19], which is in agreement with our results. Then, we identified three key genes (CXCL9, CXCR3, and ITGAX) as critical for FGR by multiple bioinformatics analyses and validated in an additional independent dataset that all three genes were highly expressed in the FGR group and had a diagnostic ability for FGR.

CXCL9 and CXCR3 are members of the chemokine family. CXCL9 is positioned on chromosome 4 in humans, which is induced by IFN-*γ* [20]. CXCR3 is a transmembrane G protein-coupled receptor, whose gene is located on chromosome Xq13 [21]. CXCR3 is the ligand for CXCL9 and also for CXCL10 and CXCL11 [22]. CXCR3 interacts with its ligands to disrupt fetal-maternal immune tolerance, triggering a range of chronic inflammatory lesions in the placenta that lead to intrauterine growth restriction, fetal death, spontaneous abortion, premature rupture of membranes, and preterm delivery [23–25]. Malaria infection during pregnancy leads to severe maternal anemia and low infant birth weight, and multivariate analysis of known predictors of birth weight suggests that elevated placental CXCL9 levels are considered an important cause of fetal growth restriction [26]. This is similar to the results of our study, where we found that the expression of CXCR3 and CXCL9 was elevated in the FGR group, and they are one of the important factors in the development of FGR.

Integrin alpha X (ITGAX) is one of the members of the integrin family, which usually acts as a receptor for the extracellular matrix. ITGAX is closely associated with tumor development, and ITGAX promotes c-Myc-mediated VEGF-A transcription by activating the PI3K/Akt pathway and binding to VEGFR2 on the cell membrane, enhancing angiogenesis during ovarian cancer growth [27]. Study to explore key genes in unexplained recurrent spontaneous abortion by targeted RNA sequencing and clinical analysis identified ITGAX as one of the immune-related genes involved in T cell activation and proliferation and cytokine receptor interactions [28]. However, there are no studies on the relationship between ITGAX and FGR. Our results suggest that ITGAX expression is elevated in FGR placental tissue and ITGAX is involved in the development of FGR, adding a new perspective to the study of the mechanisms of FGR.

Finally, we also investigated the biological functions of CXCL9, CXCR3, and ITGAX. GSEA revealed that CXCL9, CXCR3, and ITGAX could activate “hematopoietic cell lineage” and “cell adhesion molecules.” Studies have shown that cell adhesion molecules are involved in the proliferation, fusion, migration, and invasion of trophoblast during placenta formation [29], and the dysregulation of the expression of these molecules can easily lead to pathological placenta, which can cause various obstetric complications such as intrauterine growth restriction [30, 31], but the exact mechanism needs further research. CXCL9, CXCR3, and ITGAX also inhibit the “cGMP-PKG signaling pathway,” which regulates the umbilical cord circulation, and the NO-induced umbilical vein relaxation observed in growth-restricted female neonates is associated with an imbalance in the NO/cGMP pathway [32].

The current study has some limitations. We explored the pivotal genes associated with FGR and their biological functions in the GSE147776 dataset and validated the pivotal genes in the GSE24129 dataset, but we still need to validate the placental tissue by the qRT-PCR analysis method, and the regulatory mechanism of hub genes in fetal intrauterine growth restriction needs to be further investigated.

## 5. Conclusions

In this study, we used WGCNA to screen the core module and identify key genes to provide new ideas for the pathogenesis of FGR and provide potential diagnostic and therapeutic targets. We will subsequently validate the findings of this study *in vivo* and *in vitro* and elucidate the specific mechanisms of the core genes in FGR.

## Figures and Tables

**Table 1:** Information of GSE147776 and GSE24129 in GEO.

ID	GSE number	Platform	Samples		Tissue	Study type	Year	Country	Group
			Con	FGR					
1	GSE147776	GPL20844	8	7	Placenta	Expression profiling by array	2020	Mexico	Discovery cohort
2	GSE24129	GPL6244	8	8	Placenta	Expression profiling by array	2011	Japan	Validation cohort

**Table 2:** Clinical information of GSE147776 and GSE24129.

Clinical information	GSE147776		GSE24129	
	Con (*n* = 8)	FGR (*n* = 7)	Con (*n* = 8)	FGR (*n* = 8)
Maternal age (years)	30.57 ± 6.18	26 ± 1.4	31.5 ± 6.5	31.4 ± 3.7
Gestational age at birth (weeks)	38.5 ± 0.48	38.5 ± 0.48	38.1 ± 0.8	37.3 ± 1.0
Newborn weight (g)	3167 ± 30.69	2175.5 ± 241.3	2891.5 ± 309.6	1765.4 ± 483.9
Newborn length (cm)	49.21 ± 0.63	45 ± 1.75	Not available	Not available
Placental weight (g)	Not available	Not available	571.4 ± 151.0	329.4 ± 61.3

## Data Availability

The data used to support the findings of this study are available from the corresponding author upon request.
